# Supplementary material for: Controlled growth of single-crystalline metal nanowires via thermomigration across a nanoscale junction
Source: Nat Commun. 2019 Oct 2;10:4478. doi: 10.1038/s41467-019-12416-x (PMC6775085; doi:10.1038/s41467-019-12416-x)
Supplement: Supplementary file 1 — Supplementary Information [file 41467_2019_12416_MOESM1_ESM.pdf]

Supplementary Information

Controlled growth of single-crystalline metal nanowires via thermomigration across a nanoscale junction

De-Gang Xie et al.

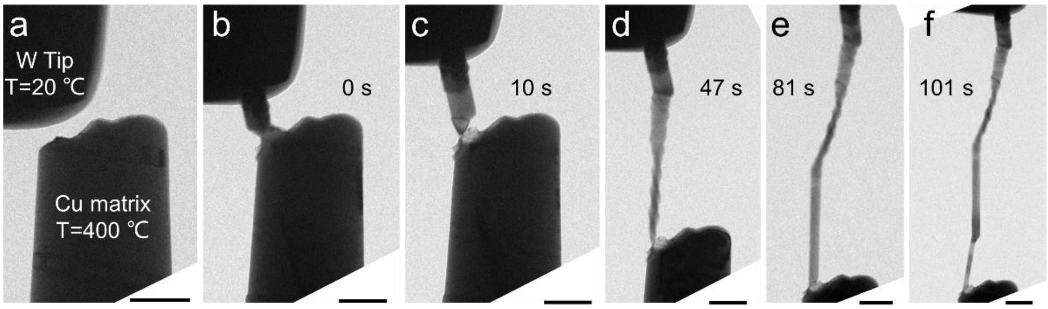

Supplementary Figure 1. The growth of a copper nanowire via thermomigration with exact experimental set-up. The Cu matrix is hold at 400 ℃(~0.50$T_{m,Cu}$). All the scale bars are 100 nm.

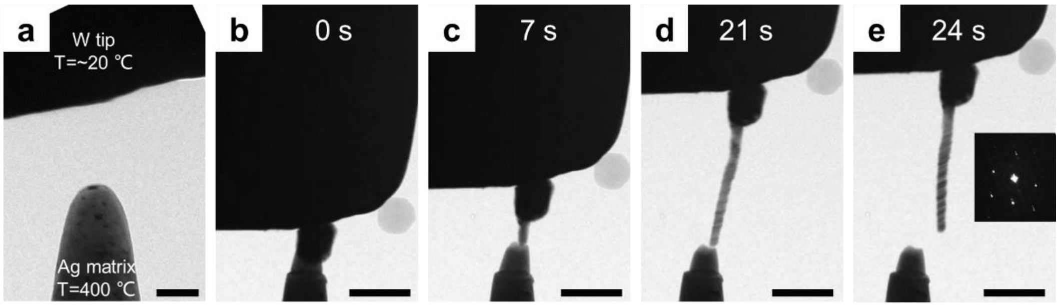

Supplementary Figure 2. The snapshots of silver nanowires grown from a Ag matrix at 400 ℃ (~0.55$T_{m,Ag}$). The scale bar in (a) is 50 nm. Scale bars in (b)-(e) represent 200 nm.

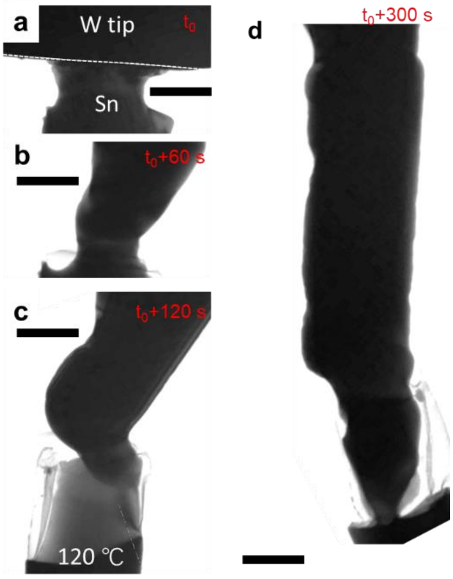

Supplementary Figure 3. The preparation of a Sn nanowire by drawing W tip at RT from the Sn substrate at 120 °C (~0.78 T_m , where T_m is the melting temperature of Sn). All scale bars are 100 nm.

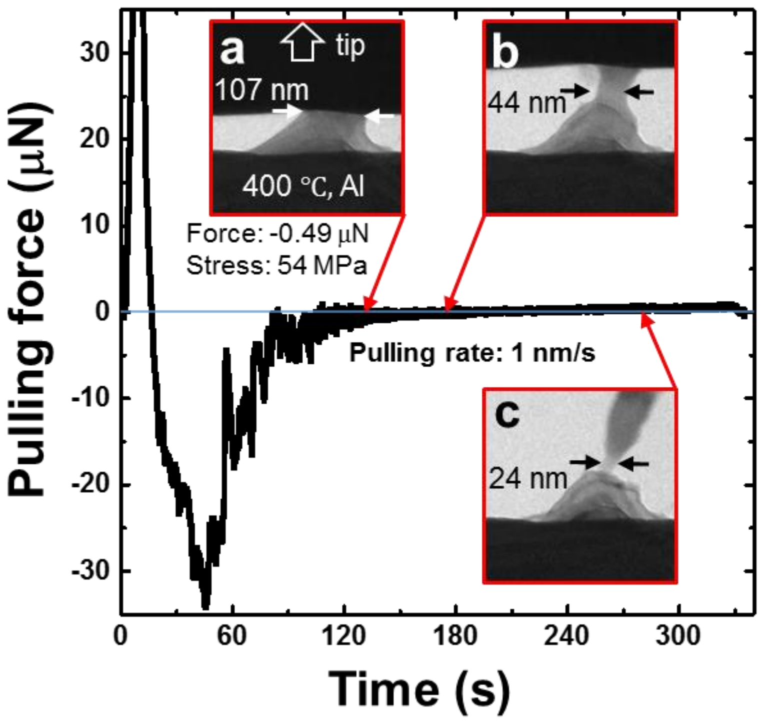

Supplementary Figure 4. Measurement of the loading stress during the hot drawing.

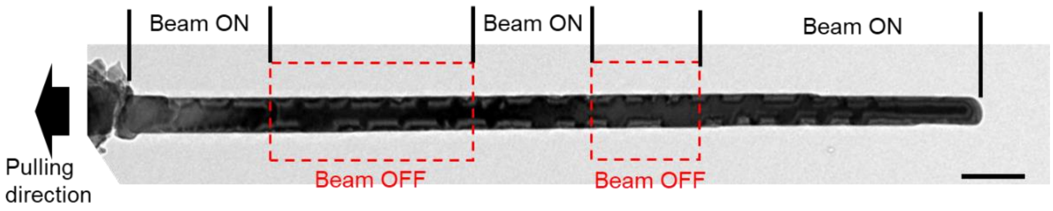

Supplementary Figure 5. A nanowire grown under both beam-ON and beam-OFF conditions. The segments grown under beam-OFF condition are labeled with dashed red rectangle. The scale bar represents 200 nm.

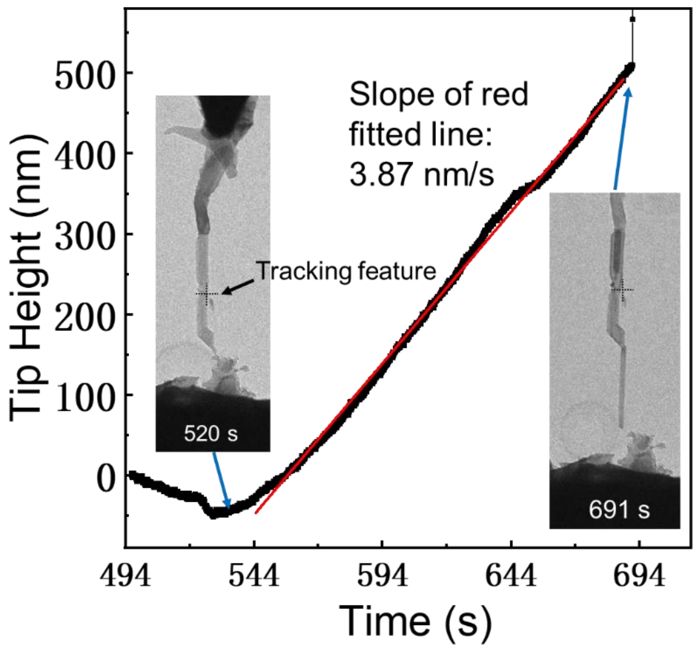

Supplementary Figure 6. Measurement of the drawing velocity in the uniform growth stage in Figure 2.

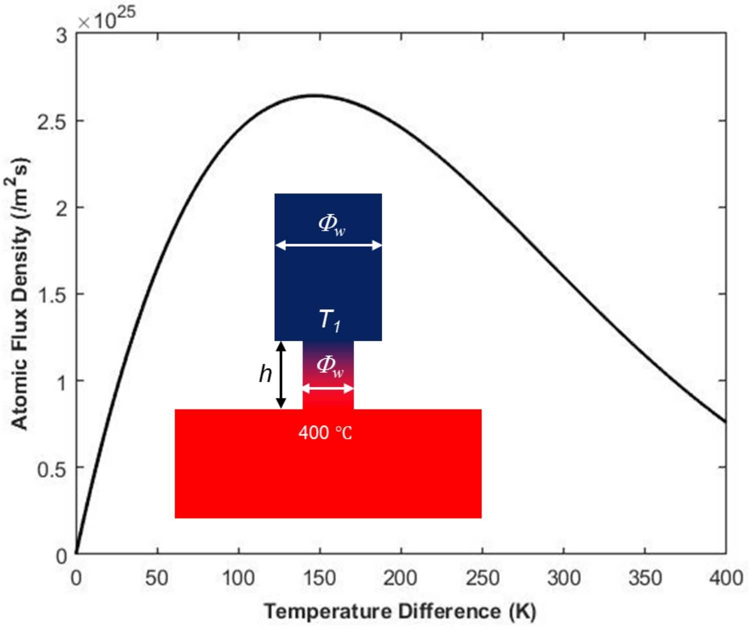

Supplementary Figure 7. Plot of atomic flux density vs. the temperature difference ΔT in the neck

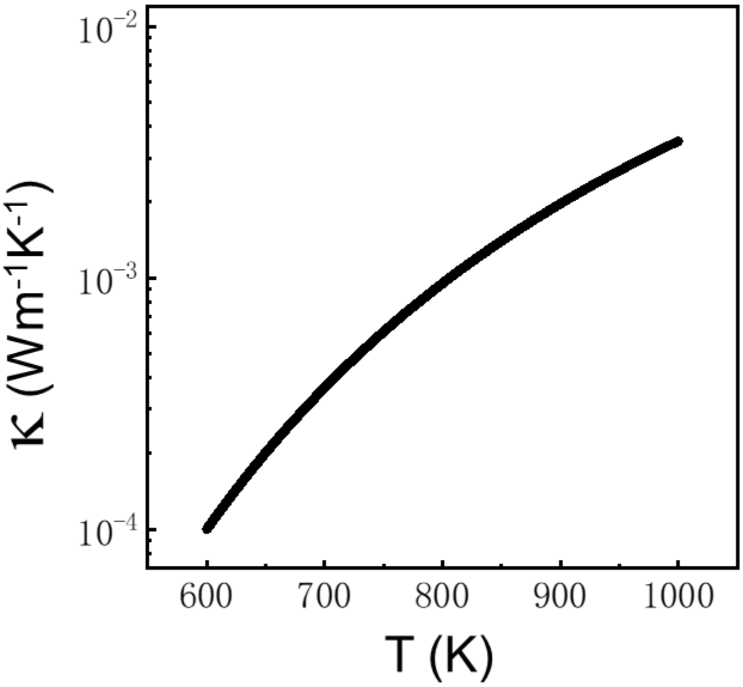

Supplementary Figure 8. The effective thermal conductivity contributed by mass transport.

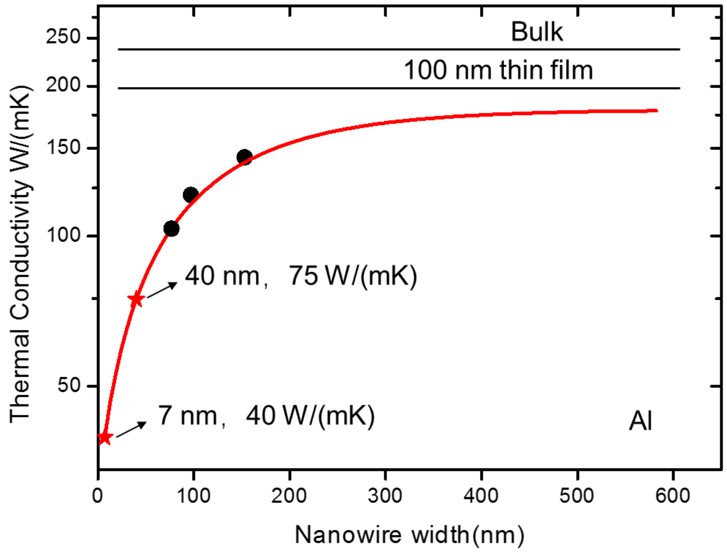

Supplementary Figure 9. The size effect of thermal conductivity in aluminum[1].

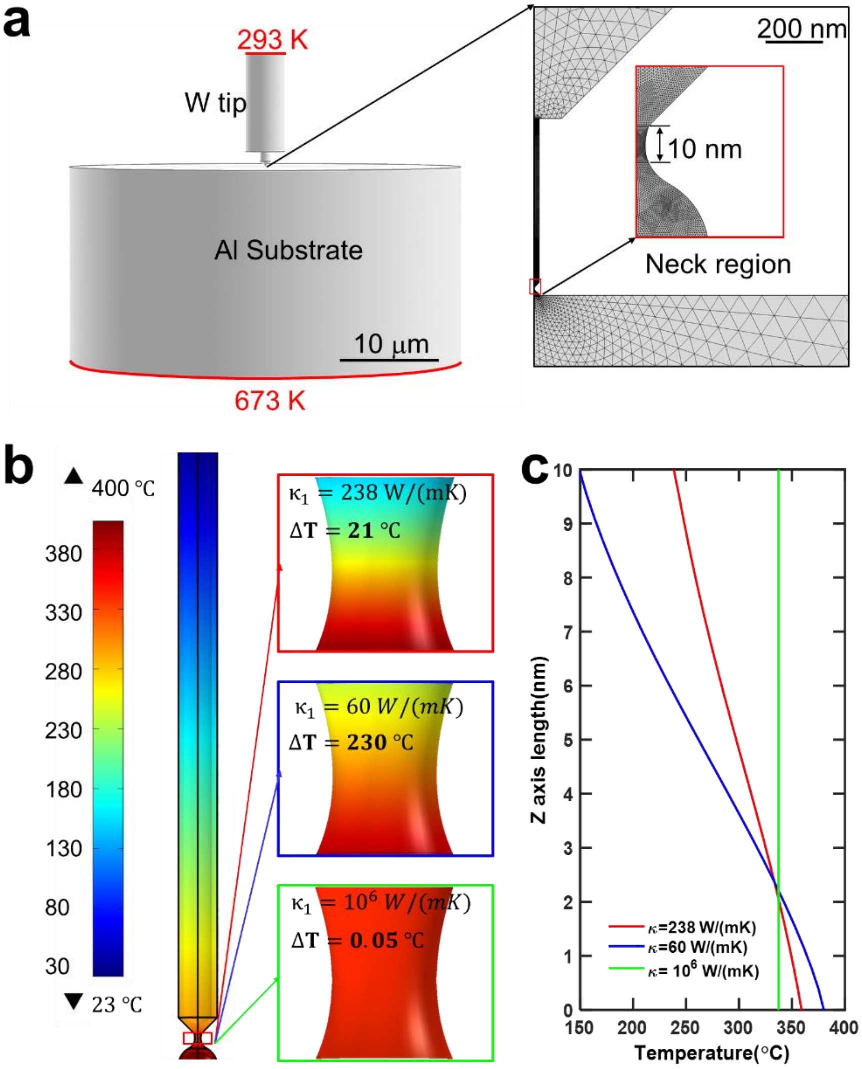

Supplementary Figure 10. FEM modeling of the temperature distribution in the neck region. (**a**) (left) Constructed geometry and boundary conditions used in this modeling. (right) The magnified view of the neck region and the mesh. The neck ligament connecting the W tip and Al substrate is finely meshed. (**b**) The simulation result of the temperature distribution in the neck region. The three inserts are results using different thermal conductivity values as indicated in the upper left corner. Also, the resultant temperature drop (ΔT) along the neck are indicated. (**c**). The temperature distribution expressed as temperature-position curve at the neck region. The red, blue, and green lines are results in neck region calculated using different κ values, that is, κ =238 W/(m·K) for bulk Aluminum, κ =60 W/(m·K) for considering size effect, and κ =1.0×10^{6}W/(m·K) for satisfying the ΔT=0.05 ℃.

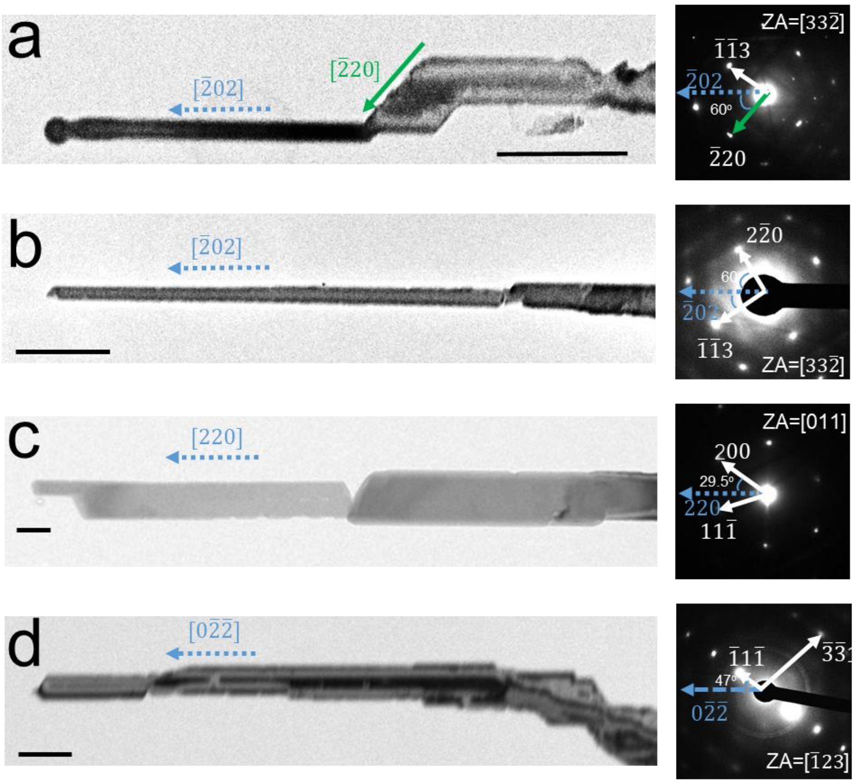

Supplementary Figure 11. Typical examples showing produced aluminum nanowires display axial orientation of <011>. All scale bars represent 200 nm.

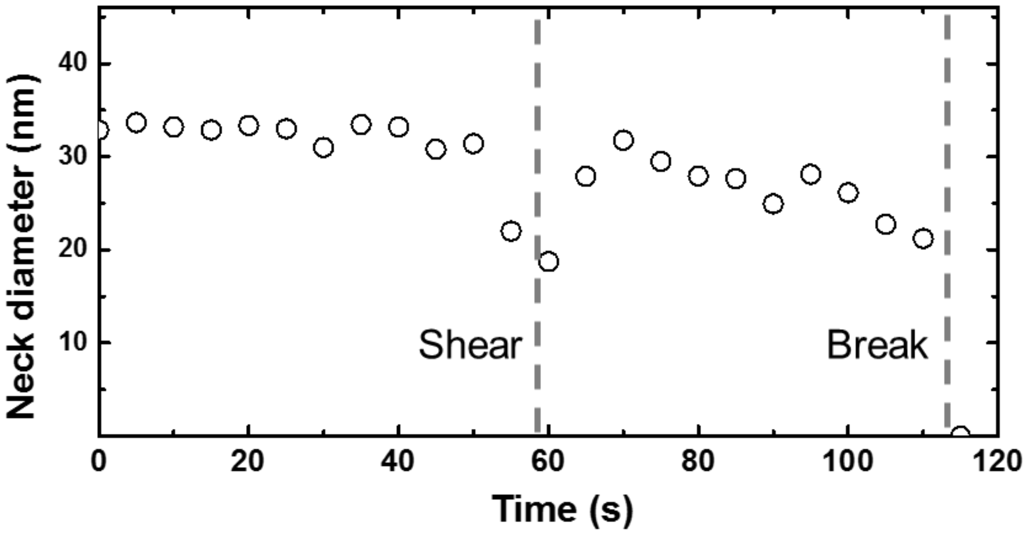

Supplementary Figure 12. The change of neck size with time for the hot drawing process in Figure 4 of the main article.

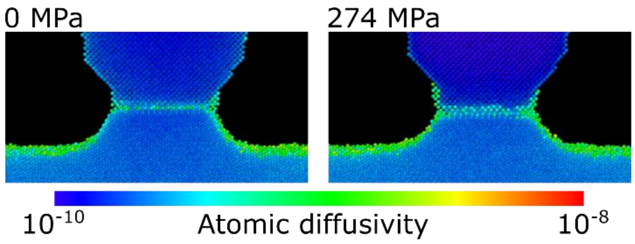

Supplementary Figure 13. Diffusivity of each atom obtained from time-averaged mean square atomic displacement.

Supplementary Note 1: Reproducibility of hot drawing experiment on Cu, Ag, and Sn

The transferability to other metal systems is also our concern. To address this concern, we have experimented with other metals including Cu, Ag and Sn, as shown in Supplementary Fig. 1-3. These results unambiguously demonstrates the good generality of our new method. Actually, this generality roots in a general physics, i.e. thermomigration which is a universal phenomenon driven by temperature gradient.

Since Cu and Ag have melting points much higher than Al, the successful repeat of the same phenomenon at 400 ℃(0.5$T_{m,Cu}$)was surprising.

Supplementary Note 2: Estimation of the shear strength of the neck-shaped ligament

As illustrated in Figure 2g, the breakup of the neck ligament and the spring-back of the nanowire process from a bent profile can be used to estimate the shear strength of the neck. By assuming that the nanowire is a round bar with a fixed upper end and the lower end is loaded with shear force in the neck, the shear strength τ of the neck region[2] can be calculated

$$\tau = \frac{3Eyr^4}{4r_n^2 l^3} \quad (1)$$

where E is the elastic modulus (70 GPa), y is the bending deflection (~120 nm), r=20 nm and l=620 nm are the radius and length of the nanowire, and r_n=3.5 nm is the radius of the neck in its thinnest region. The shear strength τ is then calculated to be 345 MPa, indicating that the neck is still strong solid.

Supplementary Note 3: Estimation of the pulling stress inside the neck-shaped ligament

A high-sensitivity force sensor (Hysitron PI95 TEM Picoindenter) was applied to measure the pulling force. The results shown in Supplementary Fig. 4 indicate when the ligament was strained to elongate, the pulling force was measured to be close to the noise floor of the sensor. Inserts (a-c) in Supplementary Fig. 4 are extracted from different moments during the ligament elongation. Based on the ligament shape with an apparent diameter of 107 nm in insert (a), the pulling force by averaging points within ± 1 s is calculated to be -0.49 μN, which corresponds to a uniaxial tensile stress σ=54 MPa in the neck/nanowire. Such a low tensile stress indicates that tensile stress shall not play a dominant role in the growth of the nanowire, and in this experiment the pulling tip mainly acted as a heat sink for setting up the temperature gradient.

Supplementary Note 4: Electron beam effect on the nanowire growth

We tried to turn off the electron beam while an aluminum nanowire is growing, to check whether electron beam will change or even stop the growth process. The results showed that nanowire growth wasn't interrupted, and the segments without electron beam illumination showed no appreciable difference with those under beam illumination, as shown in Supplementary Fig. 5. This is a solid evidence that electron beam effect has negligible effect on the growth of nanowire under our experimental condition.

Supplementary Note 5: The simplified geometrical model for estimating the atomic flux driven by thermomigration

The surface diffusivity[3,4] of aluminum is

$$D_s = 0.014\exp\left(\frac{-6.54T_m}{T}\right)\text{cm}^2/\text{s} \tag{2}$$

Assuming that the temperature in the neck region is linearly distributed from the hot bottom with temperature = 400 ℃ to the cold top with temperature T_1, as shown in the simplified model in Supplementary Fig. 7, we have a temperature difference $\Delta T=400-T_1$, and then both the temperature gradient ∇T and the average temperature T in the neck can be expressed in terms of ΔT,

$$\nabla T = \Delta T \ / h \tag{3}$$

$$T = 400 - \Delta T / 2 \tag{4}$$

Substituting Equations (2-4) into Eq. (2), J_T can also be expresses as a function of ΔT

$$J_T = \frac{-1.7\times 10^{33}}{(673-\Delta T/2)^2} e^{\left(\frac{-6101.8}{673-\Delta T/2}\right)} \Delta T \tag{5}$$

The J_T vs. ΔT plot is shown in Supplementary Fig. 7.

Supplementary Note 6: Theoretical calculation of the effective thermal conductivity due to surface mass transport

The real scenario could be very complicated. Besides temperature, stress gradient and chemistry gradient can all drive the creep process though the surface diffusion channel.

Obviously on the neck surface there exists a large flux of atoms, which can also carry heat from the hot to the cold. This additional source of heat transport may also contribute to the effective thermal conduction. The mass flux can be written as

$$J_m = \frac{cD}{kT}\nabla(Q\ln T) \tag{6}$$

where c is atomic concentration (=1×10^{29}/m^3), D/kT is mobility, and $lnTQ$ is the driving force.

The energy flux are then can be represented as

$$J_E = \frac{cD}{kT}\nabla\left(Q\ln T\right)Q = \frac{cDQ^2}{kT^2}\nabla T \tag{7}$$

The effective thermal conductivity due to mass transport is

$$\kappa = \frac{cDQ^2}{kT^2} \tag{8}$$

By plugging in numbers of Q=0.07 eV, k=8.6×10^{-5} eV/K, and D-T relation in equation (8) in the main text, we can plot κ versus T as shown in Supplementary Fig 8 below. The effective thermal conductivity, k, is indeed small compared to the bulk value (10^2 W/m/K) so that this part is negligible for explaining the thermomigration.

Supplementary Note 7: <u>Size-dependent thermal conductivity at the neck and nanowire</u>

Thermal conduction in solids is contributed by electrons and phonons. Bulk aluminum has a high thermal conductivity of κ>200 Wm^{-1}K^{-1}, mostly from electrons. However, when the geometrical size approaches the electron mean free path (λ_e=~22 nm near RT)[5] or the phonon mean free path (λ_{ph}=~5-7 nm near RT)[6,7], the thermal conductivity is reported to decrease quickly. To our knowledge, there is no reported experimental measurement of thermal conductivity to establish the relation between κ and size near λ_{ph} or λ_e. N. Stojanovic et al.[1] directly measured the κ of thin aluminum belts with 100 nm thickness and 75 nm width to be 100 W/m·K, approximately 0.4 of bulk value (235 W/m·K). By extending the measured κ vs. size relation[1], the thermal conductivity of aluminum wire is κ_{neck}=~40 W/m·K for cross section of 7 nm × 100 nm, and κ_{nw}=75 W/m·K for 40 nm × 100 nm, as shown in Supplementary Fig. 9. These two numbers,

though larger than the true number, will be used in the following FEM modeling for estimating temperature distribution in the experiment.

Supplementary Note 8: Temperature distribution by FEM modeling

Commercial FEM software COMSOL was used in the modeling. A 2D axisymmetric geometrical model was constructed to mimic region within a few hundreds of microns from the growing site. Geometrical parameters of the nanowire and the neck ligament are chosen to represent the real one shown in Figure 2. For nanowire, the diameter and length are 40 nm and 600 nm separately, and for the neck-shaped ligament, the length is 10 nm and the thinnest part has a diameter of ~7 nm. Supplementary Fig. 10a shows the meshed model. The heat source is set to be the bottom surface of the aluminum substrate, which is at 400 °C, while the heat sink locates at the top surface tungsten rod, which keeps at room temperature. However, due to the scale limit of the geometry, the distance between the heat source and heat sink is 315 microns, much smaller than the real centimeter scale distance. In this finite element model, only heat conduction is considered, because the experiment was carried out in vacuum and the radiative transfer was negligible at the experimental temperature.

Simulation results using bulk thermal conductivity of aluminum were shown in Supplementary Fig. 10b. The steepest temperature drop is found in the neck region, with a ~200 K drop from ~670 K at the bottom to ~470 K at the neck top, corresponding to a temperature gradient $\nabla T_1 = 10^{10}$ K/m, 3 orders of magnitude higher than the required number estimated in the main article.

Also, we performed simulation using reduced thermal conductivity κ considering the size effect, that it, 40 W/m·K at the neck region and 75 W/(m·K) at the aluminum nanowire. The temperature distribution at the neck region was shown in the Supplementary Fig. 10c. The lower thermal conductivity produces a steeper temperature distribution in the neck and nanowire, with the a highest temperature

gradient ∇T_2= ~2 × 10^{10} K/m at the neck region. If the temperature gradient ∇T_2 is substituted into equation (2) in the main article, the estimated atomic flux will be J=~10^{25} $m^{-2}s^{-1}$, which is 3 orders of magnitude larger than what we observed in the experiment.

Since there is a ∇T gap of 4 orders of magnitude, we are curious to know that how much apparent thermal conductivity κ_{neck} at the neck region will lead to the estimated small ∇T=1.3×10^6 K/m. By trying different κ_{neck} at the neck region in the FEM model, the κ value at the neck region shall be as high as κ_{neck}=~10^5 W/(m·K), also shown in Supplementary Fig. 10c. This value is unrealistically high, indicating that there must be other process other than pure thermal conduction in the neck region.

Supplementary Note 9: <u>Calculation of diffusivity for each atom using time-averaged mean square atomic displacement</u>

The time-averaged mean square atomic displacement of each atom i was calculated as follows,

$$\overline{\delta \boldsymbol{r}_i^2}(\Delta t;t) = \frac{1}{t-\Delta t}\int_0^{t-\Delta t}\left[\boldsymbol{r}_i(t'+\Delta t)-\boldsymbol{r}_i(t')\right]^2 dt' \qquad (9)$$

where $\boldsymbol{r}_i$, $t = 10$ ns and $\Delta t = 0.01$ ns are atomic position of atom i, duration time (simulation time), and lag time (observation interval), respectively. Thus the diffusivity of each atom, D_i, was obtained as

$$D_i = \frac{\overline{\delta \boldsymbol{r}_i^2}}{6t} \qquad (10)$$

The result is shown in Supplementary Fig. 13

Supplementary references

1 Stojanovic, N., Maithripala, D. H. S., Berg, J. M. & Holtz, M. Thermal conductivity in metallic nanostructures at high temperature: Electrons, phonons, and the Wiedemann-Franz law. *Physical Review B Condensed Matter* **82**, 2283-2288 (2010).

2 Nilsson, S. G., Borrise, X. & Montelius, L. Size effect on Young's modulus of thin chromium cantilevers. *Appl. Phys. Lett.* **85**, 3555-3557 (2004).

3 Gjostein, N. A. in *Diffusion* (ed H. I. Aaronson) Ch. 9, 241-274 (American Sociaty for Metals, 1973).

4 Tu, K. N. in *Solder Joint Technology* Vol. 117 *Springer Series in Materials Science* Ch. 8, 211-243 (Springer New York, 2007).

5 Hanaoka, Y., Hinode, K., Takeda, K. I. & Kodama, D. Increase in electrical resistivity of copper and aluminum fine lines. *Mater. Trans.* **43**, 1621-1623 (2002).

6 Zhou, Y., Anglin, B. & Strachan, A. Phonon thermal conductivity in nanolaminated composite metals via molecular dynamics. *J. Chem. Phys.* **127**, 184702 (2007).

7 Majumdar, A. & Reddy, P. Role of electron–phonon coupling in thermal conductance of metal–nonmetal interfaces. *Appl. Phys. Lett.* **84**, 4768-4770 (2004).
